# UNDERSTANDING THE HOME-BASED SERVICE NEEDS FOR OLDER ADULTS: A SCOPING REVIEW FROM CHINA

**DOI:** 10.1093/geroni/igae098.2962

**Published:** 2024-12-31

**Authors:** Hengyuan Zhang, Sifeng Zhang

**Affiliations:** Xi’an Jiaotong University, Xi’an, Shaanxi, China (People’s Republic); Xi’an Jiaotong University, Xi’an, Shaanxi, China (People’s Republic)

## Abstract

The range of home-based services for older adults in China haven’t been clearly defined, which adversely affects their effectiveness in enhancing well-being. It is crucial to develop a better understanding of the home-based service needs of older adults. This paper employs a scoping review methodology to explore the service needs of older adults aging in place. We searched empirical research articles from four Chinese databases, including CNKI, CQUVIP, CSDP and ChaoXing. A framework based on pecking order preference theory and demand spillover theory was established to categorize and analyze the literature findings. The final analysis includes 134 studies selected from a pool of 6,565 articles. By encoding the results of the articles, home-based services for older adults were categorized into four domains: medical and health services, family replacement services, disability care services, and individual auxiliary services. Among these, medical and health services emerge as the most urgently needed category for older adults. Family replacement services follow closely, as their benefits extend to family members. While the need for disability care services remains relatively low, they exhibit high rigidity. Individual auxiliary services, although least needed, consume significant public resources. We advocate for targeted public policies that allocate resources effectively, enhance older adults’ health, and provide care services for those with disabilities in daily living activities.

